# Can contralateral lymph-node metastases be ruled out in prostate cancer patients with only unilaterally positive prostate biopsy?

**DOI:** 10.1007/s10147-023-02407-w

**Published:** 2023-09-07

**Authors:** Bianca Michalik, Svenja Engels, Leonie Kampmeier, Lena Dirks, R.-Peter Henke, Friedhelm Wawroschek, Alexander Winter

**Affiliations:** 1 University Hospital for Urology, Klinikum Oldenburg, Department of Human Medicine, School of Medicine and Health Sciences, Carl von Ossietzky University Oldenburg, Rahel-Straus-Str. 10, 26133 Oldenburg, Germany; 2 Institute of Pathology Oldenburg, 26122 Oldenburg, Germany

**Keywords:** Prostate cancer, Lymphadenectomy, Biopsy, Metastases, Sentinel lymph node

## Abstract

**Purpose:**

Our study evaluated the diagnostic benefits of bilateral pelvic lymphadenectomy in prostate cancer patients with unilaterally positive prostate biopsy.

**Methods:**

Our retrospective analysis included clinical, surgical, and histopathological data of 440 prostate cancer patients treated with radical prostatectomy and bilateral sentinel-guided and risk-adapted complementary extended pelvic lymphadenectomy at our hospital between 2015 and 2022. We performed multiparametric logistic regression analysis to identify the most relevant predictive factors for detecting lymph-node metastasis in this group of patients.

**Results:**

Overall, 373 patients (85%) had histopathologically bilateral tumours and 45 (10%) pN1 status, of which 22 (49%) also had lymph-node metastasis contralateral to the side of the positive prostate biopsy. In two patients with confirmed unilateral disease in prostatectomy specimens, bilateral lymph-node metastases were observed. Eight pN1 patients would have been missed by unilateral pelvic lymphadenectomy, resulting in a false-negative rate of 18%, 82% sensitivity, and 98% accuracy. Clinical tumour category, International Society of Urological Pathology grade, and percentage of prostate biopsy cores that are positive, as well as number of dissected lymph nodes contralateral to positive prostate biopsy, were determined as the most relevant predictive factors for detecting lymph-node metastasis. Our analysis was limited by its retrospective nature as well as by the fact that 80% of the patients did not receive MRI-targeted biopsy.

**Conclusion:**

Our study highlights the diagnostic value of bilateral pelvic lymphadenectomy and the need for careful planning in surgery for prostate cancer patients with unilaterally positive prostate biopsy.

**Supplementary Information:**

The online version contains supplementary material available at 10.1007/s10147-023-02407-w.

## Introduction

In prostate cancer, lymph-node status is a relevant prognostic factor for oncological outcome and adjuvant therapy planning [1]. Despite the rise in new imaging technologies, such as ^68^ Ga-prostate-specific membrane antigen (PSMA) positron emission tomography/computed tomography (PET/CT) [2], pelvic lymphadenectomy currently remains the most accurate lymph-node staging procedure and is recommended by the international guidelines for patients undergoing radical prostatectomy in a risk-adapted fashion [3]. Because of its invasiveness and its association with intra- or post-operative complications, as well as additional morbidity of patients [4], the therapeutic benefit of pelvic lymphadenectomy is still questioned [1]. To reduce overtreatment and potential adverse effects associated with pelvic lymphadenectomy, numerous nomograms have been developed for predicting individual risk of lymph-node metastasis according to clinical parameters [5–7]. Nonetheless, guideline adherence is remarkably low [8], and there is no consensus about the anatomical extent of pelvic lymphadenectomy [1]. One reason might be that the lymphatic drainage pattern of the prostate is rather complex and highly variable between individual patients [9, 10], and therefore, the number of dissected lymph nodes must be high to ensure proper staging [11, 12]. Several studies indicated that unilateral prostate cancer might preferentially spread ipsilaterally [13, 14]. In this context, the question arose as to whether prostate cancer patients with unilaterally positive prostate biopsy could be spared bilateral pelvic lymphadenectomy. Data presented in the previous studies investigating the predictive ability of prostate biopsy on the side of lymph-node metastasis in prostate cancer patients are somewhat heterogeneous, which might hinder direct comparisons [13, 15–18]. Therefore, the aim of our study was to evaluate the diagnostic value of bilateral pelvic lymphadenectomy in prostate cancer patients with unilaterally positive prostate biopsy.

## Patients and methods

### Patient population

**Fig. 1:**
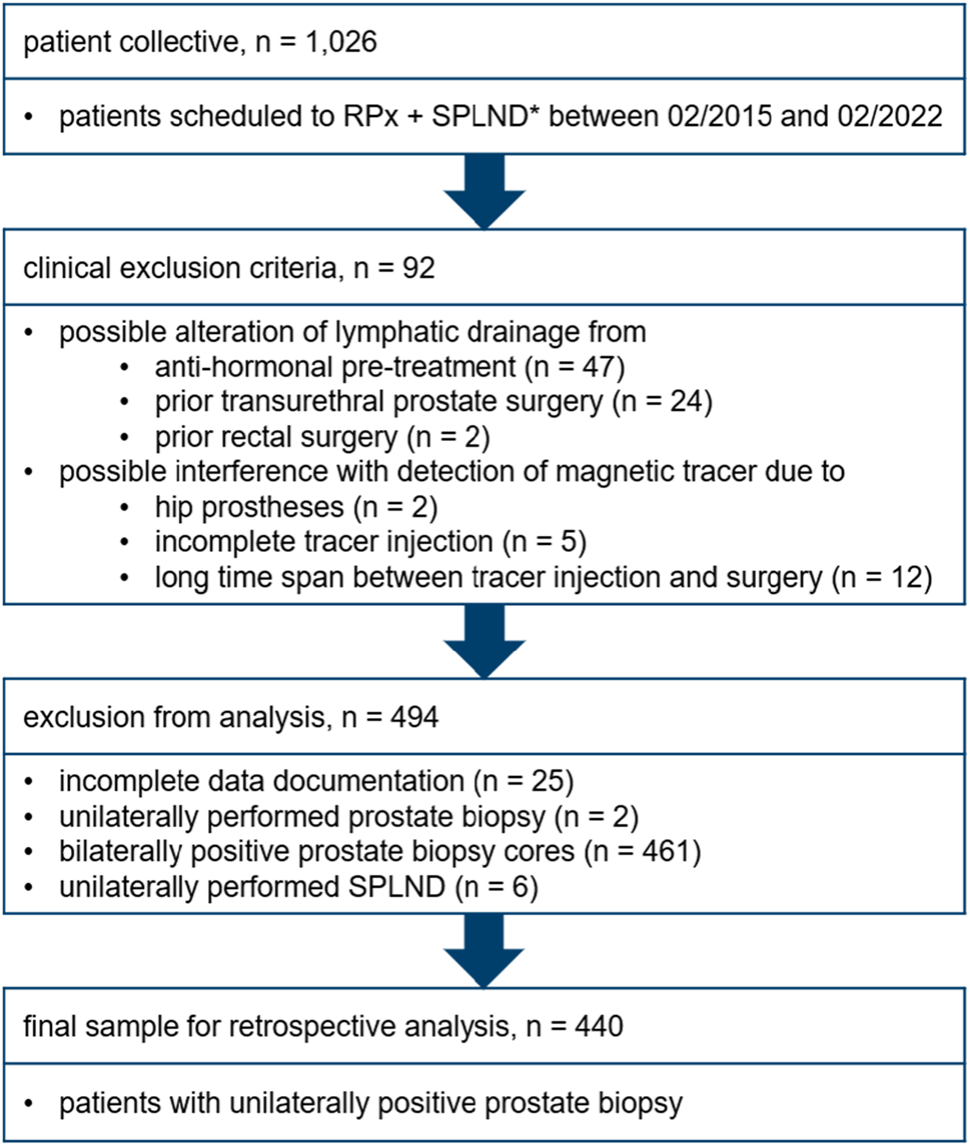
Flowchart of data validation process and exclusion criteria applied to the original patient collective. *Sentinel pelvic lymph-node dissection (SPLND) was performed at an individual risk of nodal involvement of ≥ 5% according to our nomogram [6] or at individual patient preference. *RPx* radical prostatectomy

Between February 2015 and February 2022, we documented 1,026 consecutive prostate cancer patients who underwent open retropubic radical prostatectomy combined with magnetometer-guided sentinel pelvic lymphadenectomy at our hospital. Patients were scheduled to sentinel pelvic lymphadenectomy at an individual risk of harbouring lymph-node metastases of ≥ 5% according to our nomogram [6] or at individual patient preference. Sentinel pelvic lymphadenectomy is a routine procedure at our hospital due to its high diagnostic accuracy and decreased risk of associated morbidity [19]. Figure 1 illustrates the data validation process as well as exclusion criteria applied to the original patient collective. The final sample for retrospective analysis included 440 patients with unilaterally positive prostate biopsy cores. The day before surgery, we informed all patients verbally and in writing about the open retropubic radical prostatectomy and sentinel pelvic lymphadenectomy, and all signed a consent form. This study was conducted in accordance with the Declaration of Helsinki and received ethical approval from the Medical Ethics Committee of the University of Oldenburg, Germany (02/06/2021, reference: 2018–140).

### Surgical procedure and histopathological examination

The vast majority (80%) of included patients underwent transrectal biopsy of the prostate by urologists in private practice or at other urological hospitals. Biopsies were performed transrectally under ultrasound guidance either systematically or targeted using magnetic resonance imaging/transrectal ultrasound fusion approximately 2 months before radical prostatectomy (median, 62 days; interquartile range, 47‒82 days). Median 12 cores were taken (range, 5 − 28 cores). The day before radical prostatectomy, all patients received transrectal, ultrasound-guided injection of superparamagnetic iron oxide nanoparticles into both lobes of the prostate to preferably map the lymphatic drainage of the whole prostate [20]. During radical prostatectomy, sentinel lymph nodes were detected by a handheld magnetometer probe and selectively removed, and pelvic lymphadenectomy was subsequently extended bilaterally along the anatomic template described by Weingärtner et al. [11] in a risk-adapted way according to our nomogram [6].

After surgery, all dissected tissue samples were formalin-fixed for approximately 24 h and routinely processed. Before cutting, radical prostatectomy specimens were colour ink marked for left/right as well as for dorsal/ventral differentiation under the microscope. Each tissue sample was cut into 2‒8 mm transverse slices and embedded into paraffin. Then, 4‒5 µm sections were stained with haematoxylin–eosin and microscopically analysed for tumour infiltration by a pathologist experienced in uropathology.

### Data analysis

Statistical analyses were performed using R 4.2.1 software [21]. Multivariate logistic regression models were calculated to evaluate a possible influence of the side of lymph-node surgery on the overall identification of lymph-node metastasis. Test predictors of the original model were the most relevant clinical parameters, i.e., prostate-specific antigen level, clinical tumour category, International Society of Urological Pathology (ISUP) grade, and percentage of biopsy cores that are positive, as derived from our nomogram [6], as well as surgical parameters, i.e., number of dissected lymph nodes and number of lymph nodes dissected contralateral to the side of positive biopsy. To make our results comparable with other studies [5 − 7], we chose overall lymph-node metastasis as predicting outcome and decided not to pool clinical tumour categories or ISUP grades. Estimates of the coefficients for the original model are provided in Table S1. Automated model selection according to Akaike’s information criterion (AIC) was performed using MuMIn [22]. The predictors of the final model were each tested in univariate logistic regression models. Estimates of the model coefficients were used to calculate odds ratios and their 95% confidence intervals using MASS [23]. Predictive accuracy, i.e., the area under the receiver-operating characteristics curve (AUC), was calculated for each model using ModelMetrics [24].

## Results

We analysed the data of 440 prostate cancer patients with unilaterally positive prostate biopsy cores who underwent retropubic radical prostatectomy in combination with bilateral pelvic (sentinel) lymphadenectomy at our centre between February 2015 and February 2022. Table 1 summarises the patients’ clinical and histopathological characteristics.

**Table 1:** Prostate cancer patients with unilaterally positive prostate biopsy cores who underwent radical prostatectomy combined with bilateral pelvic (sentinel) lymphadenectomy (*n* = 440). Clinical and histopathological patient characteristics

	Median (IQR)
Age [years]	67 (62‒71)
PSA [ng/ml]	8.1 (5.9‒12.0)
Time from biopsy [days]	62 (47‒82)
Positive biopsy cores [%]	22 (10‒36)
Clinical tumour category	Number of patients (%)
1c	277 (63)
2	160 (36)
3	3 (1)
Biopsy ISUP grade	Number of patients (%)
1	105 (24)
2	227 (52)
3	59 (13)
4	37 (8)
5	12 (3)
	Median (IQR)
Predicted probability of pN1* [%]	9.3 (5.4‒21.2)
Pathological tumour category	Number of patients (%)
2	300 (68)
3	139 (32)
4	1 (0)
Post-operative ISUP grade	Number of patients (%)
1	21 (5)
2	271 (61)
3	104 (24)
4	25 (6)
5	19 (4)
	Median (IQR)
Total number of dissected LNs	13 (10‒17)
Total number of dissected SLNs	7 (4‒9)
Number of contralateral LNs	6 (4‒8)
Number of contralateral SLNs	3 (1‒5)
Number of pN1 patients (%)	45 (10)
Median number of LN metastases per patient	2 (1‒3)
Median number of SLN metastases per patient	1 (1‒2)

*Probability predicted by our nomogram [6]. *ISUP* International Society of Urological Pathology, *IQR* interquartile range, *(S)LN* (sentinel) lymph node, *pN* pathological nodal status, *PSA* prostate-specific antigen

**Fig. 2:**
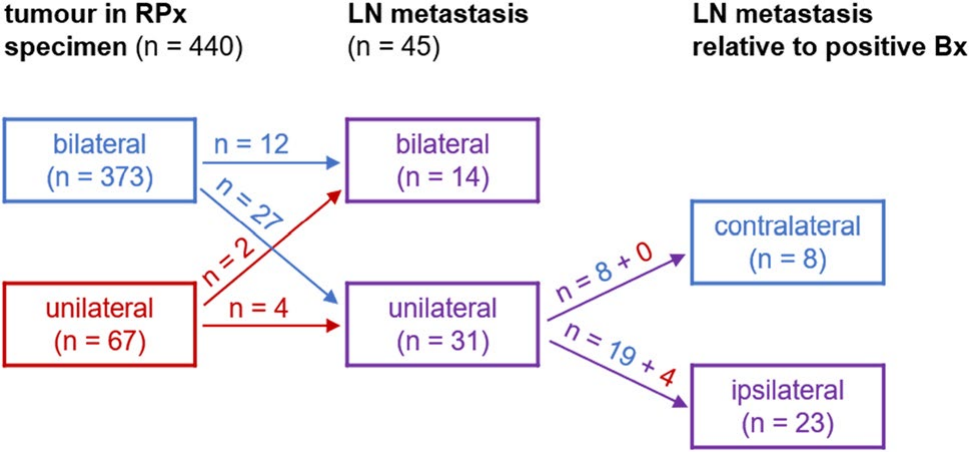
Flowchart of histopathologically confirmed tumour extent in radical prostatectomy (RPx) specimens of patients with unilaterally positive biopsy (Bx) and detected lymph-node (LN) metastases. Contralateral and ipsilateral LN metastases refer to the side of positive biopsy

In 350 patients (80%), we observed upstaging between clinical and pathological tumour categories. Tumour differentiation (ISUP grading) was matched between prostate biopsy and radical prostatectomy specimens in 253 patients (57%). Most radical prostatectomy specimens (*n* = 373, 85%) revealed bilateral tumours (Fig. 2). In only 67 patients (15%), the tumour was restricted to the side of positive prostate biopsy (indicated as unilateral in Fig. 2). Lymph-node metastases were detected in 45 patients (10%), of whom 6 patients (13%) had histopathologically confirmed unilateral tumours (Fig. 2). In 23 patients (51%), lymph-node metastasis was restricted to the side of positive prostate biopsy (indicated as ipsilateral in Fig. 2). Contralateral lymph-node metastases were detected in 22 patients (49%; Fig. 2). In two patients with bilateral lymph-node metastases, the tumour was actually restricted to the side of positive prostate biopsy (Fig. 2). In these two patients, the contralateral metastases were identified in sentinel lymph nodes. Eight pN1 patients had lymph-node metastases only contralateral to the side of positive prostate biopsy (Table 2), which resulted in a false-negative rate of 18%, because these patients would have been missed if pelvic lymphadenectomy had exclusively been performed ipsilaterally. The resulting sensitivity of unilateral pelvic lymphadenectomy was 82% and its accuracy was 98% (395 true-negative and 37 true-positive cases).

**Table 2:** Summary of results of systematic prostate biopsy and of final histopathological examination of radical prostatectomy specimens for eight cases of lymph-node metastases only contralateral to the side of positive prostate biopsy

Case	Biopsy			Final histopathology: whole RPx specimen							Final histopathology: contralateral* lesion			
	T side	ISUP grade	Positive cores [%]	P weight [g]	pT	T side	ISUP grade	T volume [ccm]	Extracapsular T extension	Seminal vesicle invasion	T side	ISUP grade	T volume [ccm]	T location
1	Right	3	33	66	3b	Bilat., mainly right	3	21	Right	Bilat	Left	1	0,5	PZ
2	Right	2	50	39	3b	Bilat., right > left	3	6	Bilat	Bilat	Left	3	2	PZ
3	Right	2	33	48	3a	Bilat., right > left	3	4,7	Right	None	Left	2	0,5	PZ
4	Left	2	42	65	3b	Bilat	2	12	Bilat	Bilat	Right	2	Right = left	TZ + PZ
5	Left	3	25	31	3a	Bilat	3	4	Bilat	None	Right	2	1	TZ + PZ
6	Right	4	38	62	3b	Bilat	3	12,3	Bilat	Bilat	Left	3	Right = left	PZ
7	Right	2	33	137	3b	Bilat., mainly right	3	12	Right	Right	Left	2	1	TZ
8	Right	5	30	127	3b	Bilat	5	20	Right	Right	Left	5	Right = left	PZ

*Contralateral refers to side of positive prostate biopsy. *Bilat.* Bilateral, *ISUP* International Society of Urological Pathology, *P* prostate, *PZ* peripheral zone, *RPx* radical prostatectomy, *T* tumour, *TZ* transitional zone

Results of systematic biopsy of the prostate and of final histopathology are summarised in Table 2 for the eight cases of lymph-node metastases only contralateral to the side of positive prostate biopsy. In all cases, histopathological examination revealed actually bilateral tumours but in about half of the cases, contralateral tumour spread was minor. Extracapsular tumour extension and/or seminal vesicle invasion, i.e., pathological tumour category > 2, was observed in all cases.

Multivariate logistic regression identified clinical tumour category, ISUP grade, percentage of biopsy cores that are positive, and number of dissected lymph nodes contralateral to the side of positive biopsy as the most relevant predictors for detecting lymph-node metastasis (Table 3). The estimates of the coefficients for the original model are detailed in Table S1. The overall predictive accuracy (AUC) of the final multivariate model was 85.3%. All identified predictors were also significantly associated with LN metastasis in the univariate analysis (Table 3).

**Table 3:** Results of univariate and multivariate logistic regression analyses predicting lymph-node metastasis in prostate cancer patients with unilaterally positive prostate biopsy (*n* = 440) based on clinical and surgical parameters

Predictor	*n*	Univariate model			Multivariate model	
		OR (95% CI)	*p*	AUC	OR (95% CI)	*p*
Clinical tumour category				72.1%		
2 vs. 1c	160	6.05 (3.04‒12.91)	< 0.001 ***		2.66 (1.20‒6.17)	0.018 *
3 vs. 1c	3	48.36 (4.33‒1089.00)	0.002 **		12.01 (0.44‒482.01)	0.159
ISUP grade				76.8%		
2 vs. 1	227	2.07 (0.65‒9.15)	0.266		1.32 (0.39‒6.00)	0.682
3 vs. 1	59	6.12 (1.74‒28.49)	0.009 **		2.41 (0.61‒12.01)	0.233
4 vs. 1	37	14.38 (4.15‒67.09)	< 0.001 ***		4.89 (1.24‒24.62)	0.032 *
5 vs. 1	12	102.00 (20.58‒714.79)	< 0.001 ***		25.16 (4.35‒194.12)	< 0.001 ***
Positive biopsy cores [%]	440	1.06 (1.04‒1.09)	< 0.001 ***	76.5%	1.04 (1.01‒1.07)	0.003 **
Number of contralateral LNs	440	1.12 (1.02‒1.22)	0.016 *	61.8%	1.10 (0.99‒1.23)	0.079

Contralateral refers to the side of positive prostate biopsy

*AUC* area under receiver-operating characteristic curve, *CI* confidence interval, *ISUP* International Society of Urological Pathology, *n* number of patients, *LN* lymph node, *OR* odds ratio

*p < 0.05; **p < 0.01; ***p < 0.001

## Discussion

This retrospective analysis of clinical as well as surgical data of prostate cancer patients with unilaterally positive biopsy revealed two main findings. First, the proportion of histopathologically verified unilateral tumours was astonishingly low (15%) and, second, the resulting false-negative rate of one-sided pelvic lymphadenectomy was correspondingly high (18%).

Our analyses confirmed the prognostic relevance of tumour load indicated by positive biopsy as a predictor of lymph-node metastasis [5–7] in patients with unilaterally positive biopsy. Nonetheless, biopsy remarkedly underestimated actual tumour expansion, which has also been observed in various other studies [25, 26]. As previous studies have already indicated, our data clearly suggest a rather limited use of biopsy data for determining the extent of lymph-node surgery in the context of radical prostatectomy [15–18].

The proportion of unilateral tumours observed in our study was much lower than that of other studies even when correcting for patient risk profile [25, 26]. Tumour progression is a rather unlikely explanation, because surgeries took place approximately 2–3 months after biopsy. Unfortunately, biopsies were performed quite heterogeneously in our analysed patient collective which is a clear limitation to the interpretation of our results. In our study, most biopsies were performed by urologists in private practice or without guidance by magnetic resonance imaging. However, our data reflect the current situation whereby magnetic resonance imaging is not yet a diagnostic standard in prostate cancer care. Furthermore, there is accumulating evidence for the underestimation of prostate cancer expansion by multiparametric magnetic resonance imaging [27].

We observed a relatively high proportion of patients with lymph-node metastases compared with other studies considering patients with unilaterally positive biopsy [16]. An obvious reason might be the likewise higher proportion of actual bilateral tumours in our data. Nonetheless, the rate of lymph-node metastasis was similar in patients with histopathologically confirmed unilateral tumours (10% vs. 9%). A more likely explanation for the relatively high proportion of lymph-node involvement in our cohort of prostate cancer patients might be the method and extent of pelvic lymphadenectomy applied by our surgeons. Median numbers of dissected lymph nodes were not higher when compared with the other studies [5, 7], which might account for more precise lymph-node surgery. We applied the sentinel node concept [9], which enables the surgeon to perform more targeted removal of pelvic lymph nodes specifically draining from the prostate [14], and might thus lead to enhanced detection of lymph-node metastases [28] while reducing the individual risk of complications and additional morbidity resulting from pelvic lymphadenectomy [28]. Higher rates of lymph-node metastasis were regularly reported in studies applying sentinel pelvic lymphadenectomy in prostate cancer patients, as reviewed recently [19].

Our data highlight the diagnostic value of bilateral pelvic lymphadenectomy in the context of radical prostatectomy. We observed a relatively high false-negative rate of pelvic lymphadenectomy restricted to the tumour-bearing side, as indicated by biopsy, which was slightly lower than observed in the previous studies [13, 15, 16, 18]. However, these studies are difficult to directly compare because of differences in their methodology, such as the studied patient collectives, the extent of pelvic lymphadenectomy, or the sample sizes.

In a recent comprehensive mapping analysis of data from 500 patients, Fujiwara et al. [17] demonstrated that the false-negative rate for detecting side-specific lymph-node metastasis decreased from 8 to 4% when adding magnetic resonance imaging-targeted biopsy to systematic biopsy. However, their analysis included only 165 patients with unilaterally positive biopsy and the rate of lymph-node involvement of this subgroup of patients was not specified. This might partly explain the generally lower false-negative rates observed in their study when compared with our data.

Our data also indicate the possibility of contralateral lymphatic drainage of the prostate, because two of our patients with histopathologically confirmed unilateral tumours had bilateral lymph-node metastases. Early lymphatic mapping studies had already suggested this possibility of lymphatic crossing [29], which was confirmed later [13, 14]. However, contralateral lymphatic drainage of the prostate is still controversial because of the overwhelming majority of ipsilateral tumour spread in prostate cancer [13, 14].

In another recent multi-institutional study, extracapsular tumour extension and/or seminal vesicle invasion, as revealed by pre-operative magnetic resonance imaging, was identified as predictive factor(s) for contralateral lymph-node metastases [30]. In line with this, the clinical tumour category is a standard predictor in classic nomograms for predicting the overall probability of lymph-node metastasis [5–7]. Despite the lack of comprehensive pre-operative imaging of the prostate, our study confirmed that all radical prostatectomy specimens of patients with contralateral lymph-node metastasis were assigned to pT > 2 categories, indicating actual extracapsular tumour extension and/or seminal vesicle invasion. Unfortunately, local imaging results are usually not intended to be used in the planning of lymphadenectomy. Preoperative (magnetic resonance) imaging currently focuses on identifying suspected tumour area(s) inside the prostate, which could be used for planning the surgical procedure, such as nerve-sparing surgery, and on detecting regional and/or distant metastases, wherein it is not very reliable. We strongly recommend standardised consideration of potential extracapsular tumour extension by pre-operative imaging in preparation for lymph-node surgery in prostate cancer patients.

## Conclusions

Our data show that unilaterally positive biopsy findings of prostate cancer do not allow conclusions to be drawn regarding the laterality of lymphatic tumour spread. Instead, our data highlight the need for careful, patient-individualised surgery planning. Further technological advancements, such as standardised pre-operative magnetic resonance imaging of the prostatic lobes, will facilitate surgical decisions.

### Supplementary Information

Below is the link to the electronic supplementary material.

Supplementary file1 (PDF 172 KB)

## Data Availability

Data analysed in this study are available from the corresponding author upon reasonable request.
