# Supplementary material for: Can contralateral lymph-node metastases be ruled out in prostate cancer patients with only unilaterally positive prostate biopsy?
Source: Int J Clin Oncol. 2023 Sep 7;28(12):1659–66. doi: 10.1007/s10147-023-02407-w (PMC10687159; doi:10.1007/s10147-023-02407-w)
Supplement: Supplementary file 1 — Supplementary file1 (PDF 172 KB) [file 10147_2023_2407_MOESM1_ESM.pdf]

Table S1. Estimates of the coefficients and Akaike's information criterion (AIC) for the original multivariate logistic regression model testing the association between lymph node metastasis and clinical as well as surgical parameters in prostate cancer patients with unilaterally positive prostate biopsy

Predictor	n	Estimate	se	z-value	p-value
Intercept		−5.480	1.269	−4.320	< 0.001 ***
ISUP grade					
2 vs. 1	227	0.344	0.680	0.506	0.613
3 vs. 1	59	0.935	0.746	1.253	0.210
4 vs. 1	37	1.562	0.751	2.081	0.037 *
5 vs. 1	12	3.250	0.957	3.397	< 0.001 ***
Clinical tumour category					
2 vs. 1c	160	0.987	0.416	2.375	0.018 *
3 vs. 1c	3	2.364	1.739	1.359	0.174
Positive biopsy cores [%]	440	0.037	0.013	2.817	0.005 **
PSA [ng/ml]	440	0.017	0.018	0.913	0.361
Number of contralateral LNs	440	0.128	0.167	0.767	0.443
Number of dissected LNs	440	−0.007	0.083	−0.088	0.930
Number of contralateral LNs * Number of dissected LNs	440	−0.001	0.008	−0.110	0.912

AIC, 234.48; ISUP, International Society of Urological Pathology; LN, lymph node; n, number; PSA, prostate-specific antigen; se, standard error.
